# Design of a general type BSSO fixation plate integrating topology and parameter optimization for various severity levels of hemifacial microsomia

**DOI:** 10.3389/fbioe.2025.1598975

**Published:** 2025-08-20

**Authors:** Chun-Ming Chang, Po-Fang Wang, Yu-Tzu Wang

**Affiliations:** ^1^National Center for Instrumentation Research, National Institutes of Applied Research, Hsinchu, Taiwan; ^2^Craniofacial Research Center, Chang Gung Memorial Hospital, Chang Gung University, Taoyuan, Taiwan; ^3^Plastic and Reconstructive Surgery, and Craniofacial Research Center, Chang Gung Memorial Hospital, Chang Gung University, Taoyuan, Taiwan; ^4^Department of Mechanical and Electro-Mechanical Engineering, TamKang University, New Taipei City, Taiwan

**Keywords:** hemifacial microsomia, BSSO, relapse, topology optimization, parameter optimization, patient-specific implant, ASTM F382

## Abstract

**Introduction:**

Patients with hemifacial microsomia exhibit varying degrees of mandibular asymmetry. The commercial plates used during bilateral sagittal split osteotomy (BSSO) surgery are often not tailored to individual patients and may lack sufficient fixation stability, increasing the risk of mandibular relapse. This study proposes a patient-specific plate design by collecting CT images of 30 cases of hemifacial microsomia to statistically analyze mandibular asymmetry.

**Methods:**

The design process integrates topology and parameter optimization analysis to develop the plate (TOPO plate), aiming for a lightweight structure with enhanced fixation stability. The structural strength and fixation stability of the TOPO plate were verified through static and dynamic four-point bending tests, as well as biomechanical testing, ensuring compliance with clinical requirements and regulatory approval for market release.

**Results:**

The TOPO plate features an asymmetrical design tailored to patient’s specific anatomy. In static/dynamic four-point bending tests, the structural strength of the TOPO plate exceeded that of commercial plates, with average proof loads of 326.9N for the right side and 389.85N for the left side. Biomechanical analysis and testing confirmed that the TOPO plate effectively limits the displacement of the mandibular segment (average 0.45mm), providing favorable fixation stability and thereby reducing the risk of relapse.

**Discussion:**

In summary, the TOPO plate is applicable for most patients with hemifacial microsomia, meeting the biomechanical requirements of the mandible and complying with market conditions. This patient-specific approach promises improved outcomes in mandibular fixation stability.

## 1 Introduction

Hemifacial microsomia (HFM) is a congenital craniofacial anomaly, second in prevalence only to cleft lip and palate (Howlader et al., 2016; Li et al., 2024). It is primarily caused by vascular disruption or hypoplasia affecting the first and second branchial arches during early embryonic development. These developmental impairments lead to a wide range of craniofacial malformations, most notably involving underdevelopment of the mandible and surrounding musculature on one side of the face. Clinical manifestations of HFM are highly variable and may include mandibular hypoplasia, facial asymmetry, malocclusion, auricular deformities, underdevelopment of facial nerves and muscles, and in more severe cases, upper airway obstruction (Howlader et al., 2016). In cases where the mandibular asymmetry is significant but the condition remains skeletal-dominant and relatively stable, surgical reconstruction using bilateral sagittal split osteotomy (BSSO) is a common and effective approach (Joss and Vassalli, 2009). BSSO involves bilateral osteotomy of the mandibular ramus, repositioning of the distal mandibular segment, and rigid fixation using plates and screws to secure the corrected position (Sato et al., 2012). However, the long-term stability of the repositioned mandible is influenced by postoperative occlusal loading, neuromuscular tension, and the extent of relapse. Relapse refers to the tendency of the mandibular segment to return toward its preoperative position, often due to muscular traction or soft tissue resistance. Several studies have confirmed that relapse can negatively impact both functional and esthetic outcomes following surgery (Joss and Vassalli, 2009; Sun et al., 2018). Therefore, achieving optimal fixation stability is critical in minimizing relapse and ensuring the long-term success of mandibular reconstruction in HFM patients.

BSSO surgery typically employs straight plates (Tseng et al., 2022), but their shape and fixation positions are ineffective in preventing hemifacial microsomia relapse (Ding et al., 2024). The author’s previous research utilized topology optimization techniques to design a customized asymmetric fixation plate addressing relapse issues in hemifacial microsomia (Wang and Wang, 2023). However, this plate design approach is considered a customized design process, where different patients require plates of varying shapes based on factors such as mandibular morphology, muscle traction forces, or occlusal forces. This lengthy design process necessitates a more refined optimization method to simplify plate design and ensure the novel fixation plate can be suitable for a broader range of patients.

The most notable methods in Structural Optimization (SO) are Topology Optimization and Parametric Optimization techniques (Abdullah et al., 2023). Each optimization approach effectively reduces material usage while maintaining essential structural strength, achieving lightweight outcomes. Topology Optimization (TO) defines optimal conditions within a fixed space, considering one or more force conditions, allowing for the removal of unnecessary materials and the design of an optimal structure (Wu et al., 2021). However, the TO structural shapes are typically irregular, and the blurred boundaries and uncertain dimension values make it challenging to define the structure in a standardized. The parametric Optimization (PO) technique selects critical dimensions within a structure to find optimal designs for each parameter based on constraints (Moradi et al., 2021). However, it is limited by the initial structural appearance, reducing design flexibility. When structural optimization techniques are applied to the design of reconstructed plates, integrating TO and OP techniques may be the ideal design procedure. The reconstructed plate’s structural design depends on the TO results, and the PO technique is further used to define critical dimensions to achieve structural optimization and lightweight design.

The trend in reconstructive plate design is to eliminate intraoperative bending through preoperative planning (Kim et al., 2023), achieving a custom plate shape that fits the bone-curved surface by metal 3D printing. 3D printing has emerged as a transformative technology in orthopedics, offering personalized solutions for complex bone defects and enhancing surgical precision. Its most prominent applications include the design and fabrication of patient-specific implants, prostheses, and surgical instruments (Brachet et al., 2023). The ability of 3D printing to precisely control scaffold architecture and material composition allows for a high degree of customization and functional optimization. This approach is particularly valuable in reconstructive surgeries following trauma, tumor resection, or congenital deformities, where conventional implants often fail to meet clinical expectations due to size mismatch, poor integration, or the risk of rejection (Prządka et al., 2025). However, it’s challenging to secure marketing approval for customized reconstructive plates. In contrast, a patient-specific plate with specific use limitations or within a defined size range is more likely to meet clinical and marketing application requirements (Premarket Notification, 2021), which is also an important design objective of this study.

The objective of this study was to create a lightweight BSSO reconstructive plate through the integration of topological and parameter optimization techniques, accommodating varying levels of patient relapse. Biomechanical analyses, static/dynamic four-point bending tests, and biomechanical tests were conducted to validate plate stability and structural strength, ensuring adherence to clinical requirements and marketing regulations.

## 2 Materials and methods

### 2.1 Medical imaging database for hemifacial microsomia with different severity levels

Considering that varying degrees of mandibular displacement correlate with different levels of relapse severity, a database of relevant mandibular dimensions had been established to assess the extent of mandibular deviation. The database comprises medical images of 30 patients diagnosed with hemifacial microsomia, including 12 males and 18 females (Protocol Title: 3D simulation and biomechanical analysis of bilateral sagittal split osteotomy in orthognathic surgery, IRB No.: 202102084B0). On each medical image of the mandible, the distance from the mandible’s condyle to the mandible’s Angle was labeled as RH, and the Angle of the mandible to the Mental protuberance was labeled as WB. The distance between the RH and the WB on each mandible was measured individually (Zhang et al., 2023), and the difference and the Root Mean Square (RMS) were subsequently calculated. The Root Mean Square (RMS) determined the extent of mandibular deviation. In this study, three cases were selected based on RMS values: medical images with maximum, minimum, and approximate mean RMS, representing minor to severe relapse of the mandibular region (Table 1).

**TABLE 1:** Measuring and analyzing the extent of mandibular deviation on 30 sets of CT images.

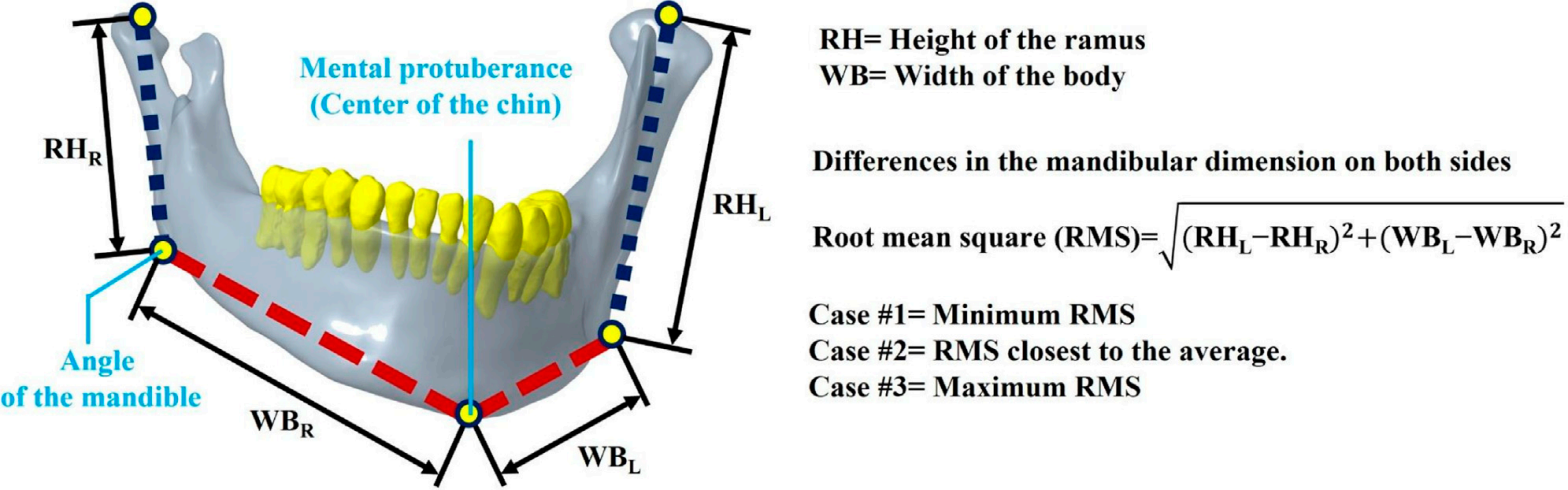						
No.	Sex	Height of the ramus	Width of the body		RMS	Case
		RH_R_ and RH_L_ differences	WB_R_ and WB_L_ differences			
1	M	23.50	10.27		25.65	
2	M	10.96	8.11		13.63	
3	F	12.52	5.60		13.72	
4	F	12.27	13.15		17.99	
5	M	15.66	7.76		17.48	
6	F	1.90	14.63		14.75	
7	M	19.89	7.26		21.17	Case #2
8	M	8.24	6.34		10.40	
9	M	11.82	3.40		12.30	
10	F	24.18	10.82		26.49	
11	F	12.23	6.78		13.98	
12	F	31.52	20.54		37.62	Case #3
13	M	15.26	3.25		15.60	
14	M	30.92	4.56		31.25	
15	F	26.66	0.28		26.66	
16	F	22.11	5.97		22.90	
17	M	9.87	24.66		26.56	
18	F	29.53	10.74		31.42	
19	F	23.16	15.38		27.80	
20	F	5.99	6.41		8.77	Case #1
21	M	10.95	13.35		17.27	
22	F	10.58	7.36		12.89	
23	F	10.30	11.09		15.14	
24	M	18.86	20.85		28.11	
25	F	12.52	1.36		12.59	
26	F	8.80	14.68		17.12	
27	F	11.23	13.86		17.84	
28	M	10.88	10.84		15.36	
29	F	12.65	4.78		13.52	
30	F	5.01	10.24		11.40	
			RMS	ave	19.97	
				sd	7.40	

### 2.2 Modeling a representative model of hemifacial microsomia and topological optimization design process

The surgeon utilized three representative mandibular models to plan the mode and distance of mandibular segment movement in each group. Subsequently, fixation plates were designed for the bilateral side of the mandibles based on this planning (Table 2). The initial plate design featured a rectangular shape (L45mm × W30mm × T1.5 mm), with bone fixation achieved using 10 screws (Ø1.5mm × L6mm). Three mandibular models, comprising cortical bone, cancellous bone, teeth, screws, and plates, were utilized as topological optimization models, with material properties assigned to each component: cortical bone (E = 13,700 MPa, ν = 0.3) (Wang and Wang, 2023; Schwartz-Dabney and Dechow, 2003), cancellous bone (E = 1,370 MPa, ν = 0.3) (Wang and Wang, 2023; Schwartz-Dabney and Dechow, 2003), teeth (E = 18,600 MPa, ν = 0.3) (Wang and Wang, 2023; Barink et al., 2003), and plates/screws (E = 110,000 MPa, ν = 0.35) (Chang et al., 2019).

**TABLE 2:** Simulating mandibular segment displacement mode during BSSO surgery and displaying the analytical formula of the topological optimization analysis.

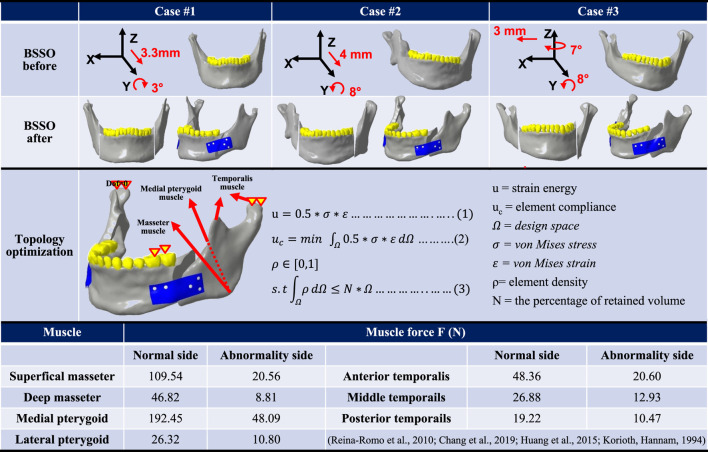

**FIGURE 1:**
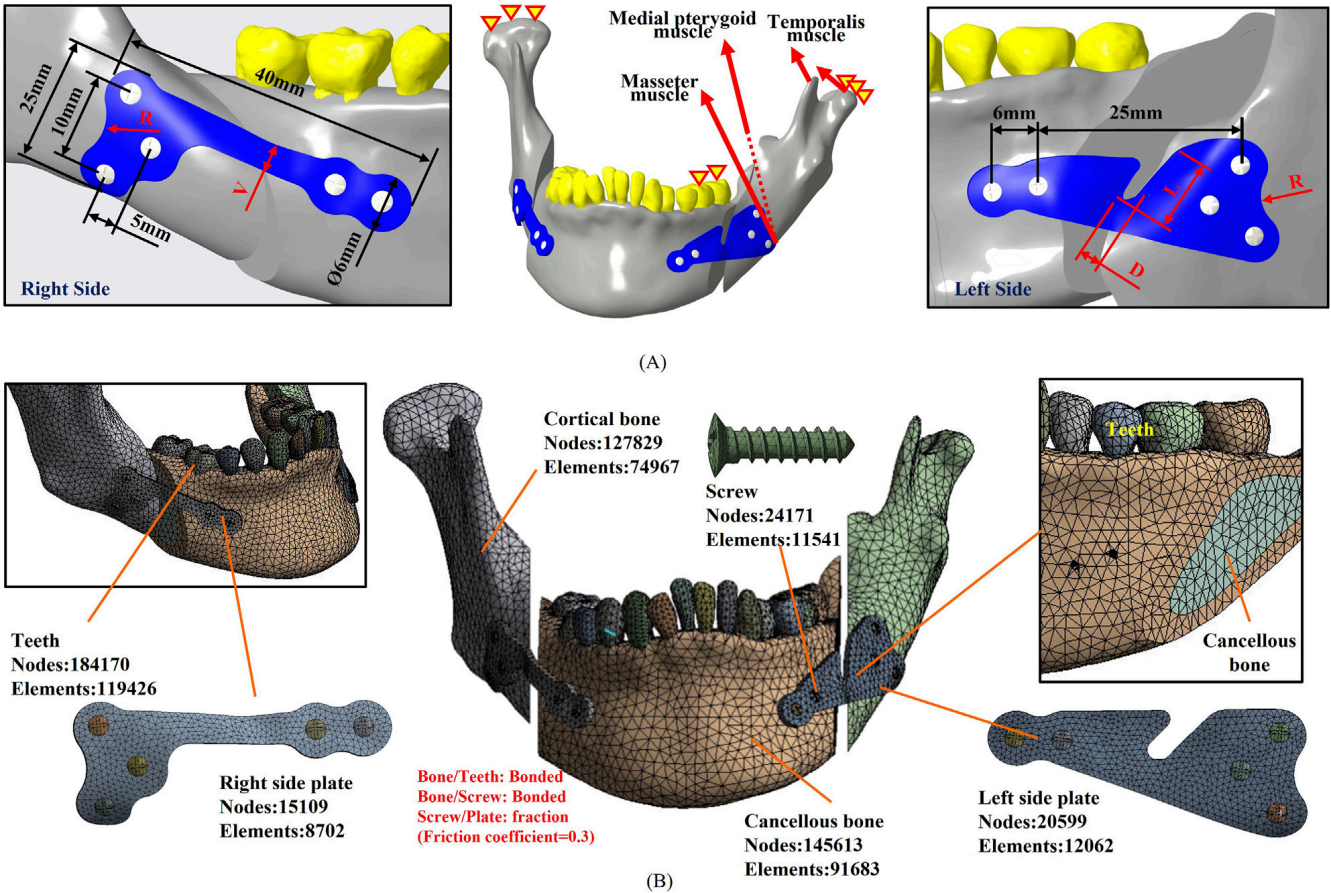
**(A)** A randomly selected case of hemifacial microsomia, not included in the primary database, was utilized as the model for parameter optimization analysis. The applied forces, boundary conditions, and parameter definitions during the analysis are presented. **(B)** Illustration of the meshed model used in the parameter optimization analysis, along with the corresponding element and node count.

The topological optimization analysis (ANSYS Workbench 2022R1, ANSYS Inc., PA, USA) incorporated asymmetric muscle traction forces (Reina-Romo et al., 2010; Chang et al., 2019; Huang et al., 2015) applied to the bilateral sides of hemifacial microsomia. This simulated muscle force relapses in the mandibular segment involve the Superficial/Deep Masseter, Medial pterygoid, Lateral pterygoid, and Anterior/Middle/Posterior Temporal muscles (Table 2). The boundary condition restricts the displacement of the unilateral first and second premolars in the direction perpendicular to the occlusal surfaces, simulating molar-food contact during unilateral occlusion. Additionally, it completely constrains the degrees of freedom in the normal direction (Z-direction) of the condylar contact surfaces of the articular socket, effectively simulating temporomandibular joint motions. The contact settings of the model simulated the postoperative bone healing state and were set as bonded between the screw and the bone and as friction (Friction coefficient = 0.3) (Wang and Wang, 2023; Naguib et al., 2024) between the plate and the screws, indicating that the sliding resistance of the two objects is proportional to the friction coefficient after force application, allowing them to be separated freely. The analysis uses 10-node tetrahedral elements for mesh planning in a free mesh. This type of element, also called a second-order tetrahedral element, can fit complex geometrical models. It increases accuracy in meshing curved surfaces and irregular geometries, ultimately allowing finite element analyses to capture complicated features with detail. The free mesh methodology guarantees adequate resolution in regions with high-stress gradients, including sharp edges, holes, or interfaces between different materials. Subsequently, the element sizes in the different geometrical models were controlled to balance computational efficiency and the accuracy of the results. Finer mesh configurations were applied in critical load-bearing regions, such as the bone plate and screws; in this case, the bone plate screws with an element size of 0.8 mm. In comparison, coarser mesh configurations were used in regions of lower mechanical significance, such as the bone with an element size of 1 mm. The number of elements and nodes for each model are presented in Figure 1B.

The topology optimization analysis procedure can be expressed by Equations 1–3 (Table 2). In this representation, the topological region corresponds to the volume of the original plate. The target function, as defined in the topological analysis, aims to minimize flexibility, with the constraint being the minimization of plate volume. The elemental density (ρ) in the original volume is a variable. After iterative computation, each elemental density is expressed as either 0 (indicating larger strain energy and the volume is deleted) or 1 (indicating smaller strain energy and the volume is kept). When the volume is deleted to the minimum while still maintaining the minimum flexibility, the iterative computation is stopped, and the optimal plate shape is obtained (Wang and Wang, 2023).

### 2.3 Parameter optimization analysis of BSSO plate based on topology results

The individual plate profiles were obtained following the topology optimization analysis of the three cases, as presented in Table 3. Each group of plates retained a similar volume and shape but differed only in dimensions. The dimensions of the plates then had to be further optimized through parametric optimization analysis. The bilateral plates were individually intersected, and the areas of overlapping volume were preserved and redrawn to achieve the smallest bilateral plate. After performing topology optimization analysis on the plates in three cases, the overlapping volumes were intersected. This intersection determines the preliminary shape of the TOPO plate, which serves as the foundation for subsequent parametric optimization. The smoothing method in analysis software, “Flatten Peaks,” was used to smooth the irregular contours of the bone plate. In this way, the topology was maintained, while local spikes on slightly rough areas were reduced, thus dealing with localized roughness, resulting in a smoother and more coherent surface profile. This approach aims at the conservation of the original volume and shape as much as possible while eliminating localized spikes.

**TABLE 3:** The intersecting plate structure after the topology optimization analysis and the parameter optimization factor settings.

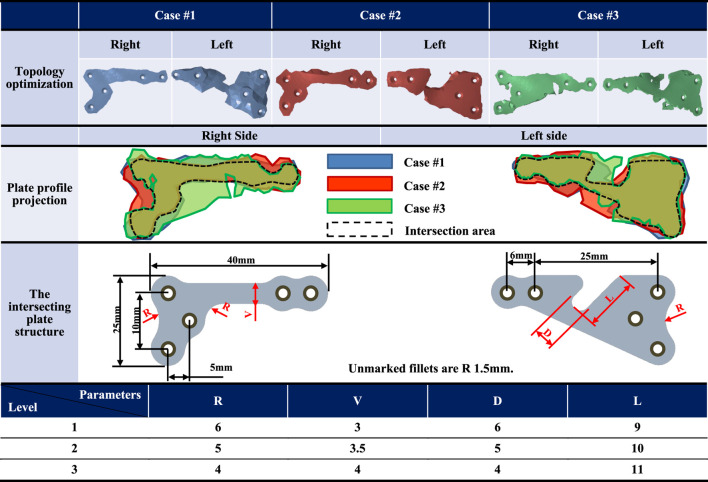

The mandibular model selected for parameter optimization analysis was derived from CASE#3, representing the scenario with the maximum displacement of the mandibular segments in the database, which is considered the worst case. This model served as the basis for analyzing intersecting plates, maintaining consistent analysis conditions as in the previous topology optimization analysis (Figure 1). The parameter optimization analysis process involved the division into parameter factors (inputs) and response definitions (outputs), selection of parameter optimization solutions, choice of Design of Experiments (DOE) methods, simulation of surfaces, and design optimization. Parametric factors were chosen to include the dimensions that could impact the structural strength of the plate. The initial positions and dimensions of the screw holes remained unchanged, and an R1.5 fillet was added at sharp edges to reduce the stress concentration. Critical geometric parameters that could influence the structural strength of the TOPO plate were considered, including the width of the right-side plate (V), the width of the left-side plate groove (D), the depth of the left-side plate groove (L), and the distal fillet on both sides of the plate (R). All these parameters were established initially based on the relative measurements of the overlapping volume (V: 3 mm, D: 6 mm, L: 9 mm, R: 6 mm) (Table 3). Subsequent modifications of these parameters were made proportionally, depending on changes in the structural strength observed for the TOPO plates.

The parameter optimization method employed in this study is the Adaptive Metamodel of Optimal Prognosis (AMOP). Given an initial Design of Experiments (DOE), AMOP automatically generates a Metamodel of Optimal Prognosis (MOP) relevant to output parameters. This method identifies areas where the metamodel is of good quality and locates where gathering additional data will increase its accuracy. Informed by this information, the AMOP autonomously conducts new simulations in subsequent iterations. This process dynamically adjusts the DOE as necessary to maximize the quality of the metamodel, requiring less manual intervention and simulations (Tyflopoulos and Steinert, 2020). Subsequently, samples were generated using the Space-Filling Latin Hypercube Sampling method, using 20 points in total. These samples were used to fit all solutions, enabling the optimization of the objective function across different variables. The dependencies among these variables were established by using regression models. Regression models for the four-parameter factors can be expressed using the polynomial formulations, as introduced in Equations 4, 5 polynomials (Tyflopoulos and Steinert, 2020; Durairaj and Gowri, 2013).

$$f\left(x\right)=\beta_{0}+\beta_{1}{\;x}_{1}+\beta_{2}{\;x}_{2}+\varepsilon \left(x\right)$$ (4)

$$f\left(x\right)=\beta_{0}+\beta_{1}{\;x}_{1}+\beta_{2}{\;x}_{2}+\beta_{11}\;x_{1}^{2}+\beta_{22}\;x_{2}^{2}+\beta_{12}{\;x}_{1}{\;x}_{2}+\varepsilon \left(x\right)$$ (5)

where:

f(x): Response of the model
x_1_, x_2_: First-order terms
x_1_^2^, x_2_^2^: Second-order terms
β_ij_: Regression coefficients
ε(x): Model error

### 2.4 Biomechanical analysis for the TOPO plate from integrated topology and parameter optimization

The TOPO plate, resulting from integrated topology and parameter optimization analyses, was subjected to biomechanical analysis to validate its stability for mandibular fixation. Using a randomly selected case of hemifacial microsomia not included in the database, biomechanical analysis was conducted under consistent mechanical and boundary conditions as the previous optimization analysis (Figure 1A). Evaluation parameters included plate stress (von Mises stress) and total displacement of the mandibular segment, confirming the stability of TOPO Plate fixation.

### 2.5 Static/dynamic four-point bending tests

The TOPO plate was manufactured using SLM metal 3D printing (AM250, Renishaw plc, Woton-under-Edge, Gloucestershire, UK) to achieve a customized fit to the mandibular surface. Regulation tests were conducted to validate the structural strength of the TOPO plate and assess its compliance with market requirements. The TOPO plate was fabricated using a commercial SLM metal 3D printer (AM250, Renishaw plc, Wotton-under-Edge, Gloucestershire, UK) with Ti6Al4V powder particles ranging in size from 15 to 53 μm. According to the manufacturer’s recommended process parameters, including a layer thickness of 30 μm, laser power of 100 W, laser exposure time of 60 μs, laser focus of 75 μm, point distance of 75 μm, and hatch distance of 20 μm, these settings are optimized to minimize internal porosity in the printed samples.

**FIGURE 2:**
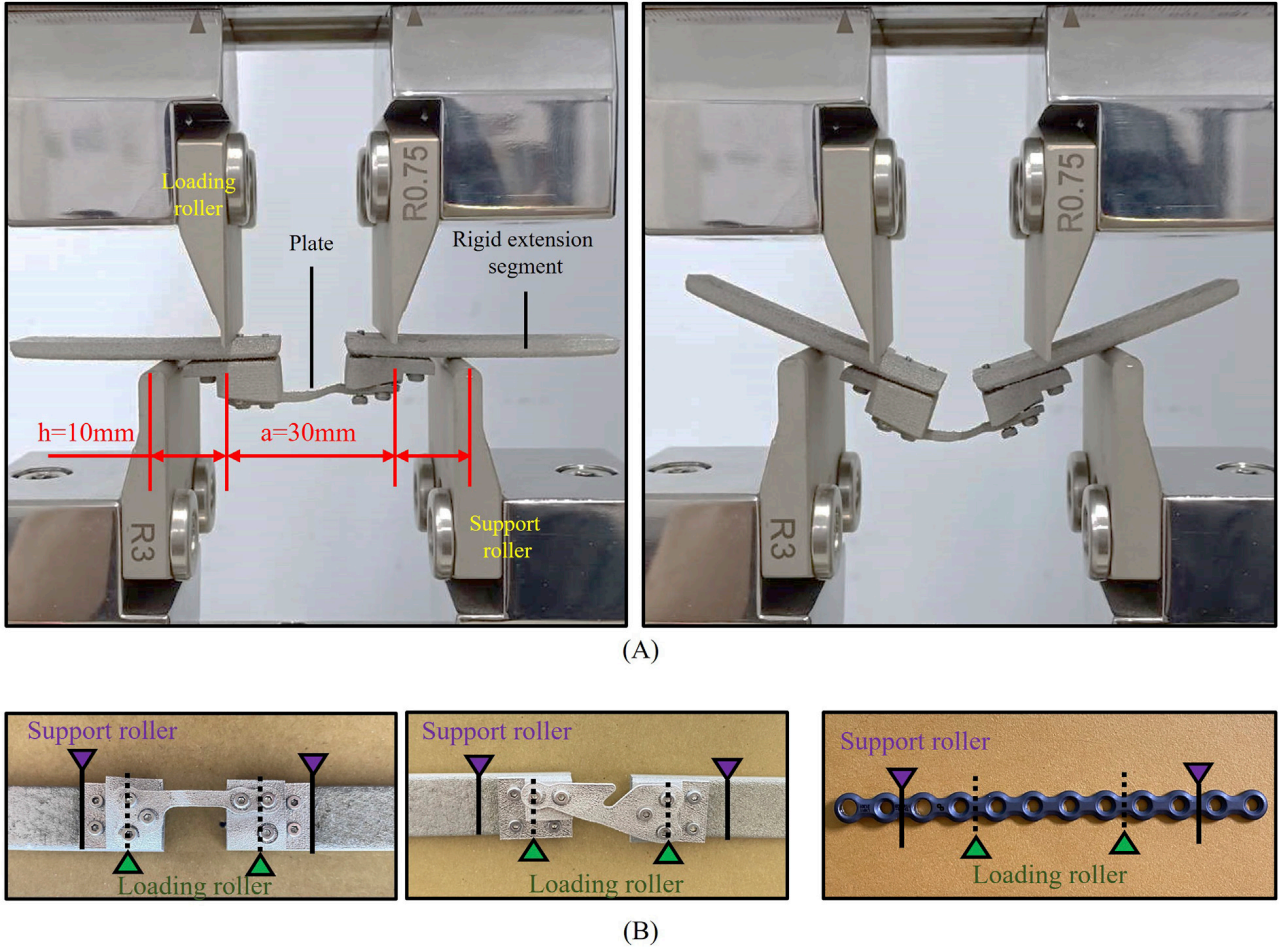
ASTM F382 regulation test. **(A)** Setup of the static/dynamic test (pre-test and post-test). **(B)** The TOPO plate is fixed with the Rigid extension segments and commercial plate.

A four-point bending test (n = 3) was conducted on both the TOPO plate and commercial plates (APLUS Biotechnology Co., Ltd., New Taipei city, Taiwan) under the ASTM F382 regulation test (ASTM F382-17 Standard Specification and Test Method for Metallic Bone Plates) (Schorler et al., 2018). The 4-point bending tests involve static and dynamic fatigue tests. (Figure 2A illustrates the two plate setup methods for the test. Due to the irregular shape and insufficient length of the plates, rigid extension segments were added to facilitate the test setup (Figure 2B). In the static four-point bending test, a loading roller applied a downward force to the rigid extension segments at 3 mm/min speed until the plate fractures. This process yielded data on the plate’s proof load, bending strength, bending stiffness, and bending structural stiffness. After completing the static four-point bending test, a load-displacement curve was generated. The linear slope of the initial section of the curve represented the bending stiffness (K). The point where the linear section deviated by 0.2% and the curve intersected indicated the proof load (P). Bending strength and bending structural stiffness were then calculated separately using Equations 6, 7.

$$\text{Bending strength}=\frac{\left(Ph\right)}{2}.$$ (6)

$$\text{Bending structural stiffness }\left(\text{EIe}\right)=\frac{\left(2h+3a\right)Kh^{2}}{12}.$$ (7)

P = the proof load
h = the loading span distance
K = the bending stiffness
a = the center span distance

The proof load obtained from the static four-point bending test served as the loading condition for the dynamic fatigue test. The TOPO plate was subjected to fatigue testing at 25%, 35%, and 45% of its proof load, while the commercial plate underwent testing at 78%, 88%, and 98% of its proof load. During the dynamic fatigue test, the plates were subjected to loading in a sine wave cycle with a frequency of 6 Hz. After 1,000,000 cycles, each plate was evaluated for any signs of plastic deformation or damage. The outcome provided insight into the fatigue strength of a plate based on the highest loading it could withstand.

### 2.6 Biomechanical testing of TOPO plate and commercial plates

To verify the stability of various bone plates for mandibular segment fixation, the biomechanical test was conducted using a single case of a hemifacial microsomia mandible model, the exact case used in previous biomechanical analyses. Since a regular artificial mandibular model could not be used for this test, the mandible model was constructed using FDM polymer 3D printing (Fortus 250MC, Stratasys Ltd., Reḥovot, Israel) (Lin et al., 2021). The mandible model was incised to simulate the BSSO surgical osteotomy, and the mandibular segments were fixed using either a TOPO plate or a commercial plate, with three samples tested for each plate type (n = 3).

**FIGURE 3:**
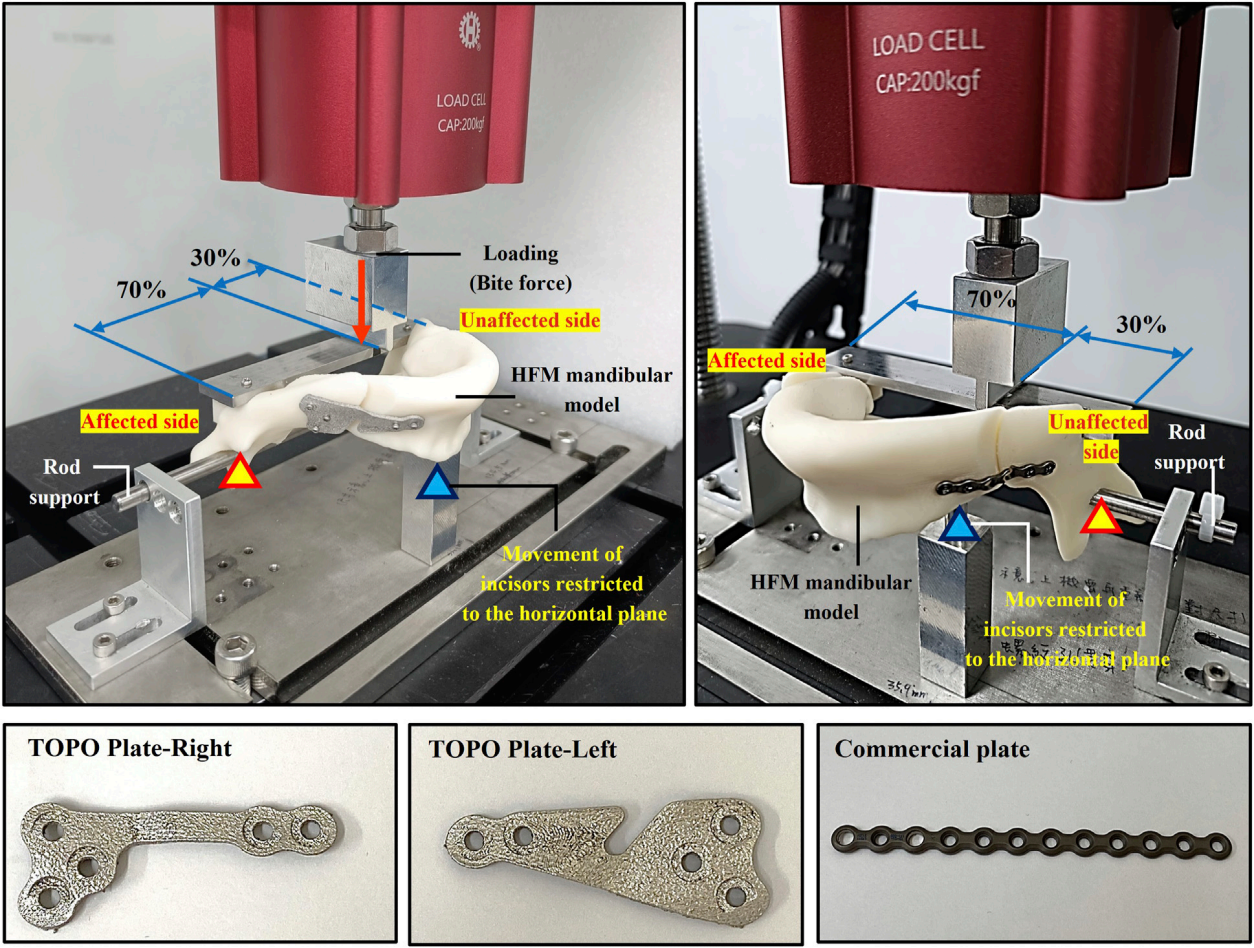
Biomechanical testing to assess the stability of different plates for mandibular segment fixation.

The biomechanical test setup involved securing the mandible with a rod through the coronoid/ramus region of the mandible, permitting rotational movement while a limit block restricted movement to the horizontal plane (Haug et al., 2001). Flat plates were positioned bilaterally at the angle of the mandible. A loading roller, connected to a dynamic testing machine (Electric servo dynamic test machine, Hung Ta Instrument Co., Ltd., Taichung, Taiwan), applied load to the mandible. The loading conditions involved distributing 70% of the load to the unaffected side (normal ramus) and 30% to the affected side (shorter ramus) (Schupp et al., 2007; Koper et al., 2021). Dynamic occlusal loading was simulated by applying a cyclic force of 20–200 N at 3 Hz for 250,000 cycles, mimicking 6 months of mandibular occlusion (Figure 3) (Lin et al., 2021). To assess the test results, fractures or loosening in the plate and screw were inspected, and the displacement of the mandibular segment post-test was measured post-test to verify the stability of fixation and resistance to relapse for various plates.

## 3 Results

### 3.1 Definition of appearance and dimensions of TOPO plate

The TOPO plate was initially designed to establish its preliminary shape through topology optimization. Subsequent parameter optimization refined the dimensions of key features to ensure optimal performance. The analysis results recommended the following dimensions: the distal fillet on both sides of the plate (R) at 5 mm, the right-side plate width (V) at 3.5 mm, the left-side plate groove width (D) at 4 mm, and the left-side plate groove depth (L) at 9 mm. These dimensions were determined to minimize the displacement of the mandibular segment, indicating that the TOPO plate combines a lightweight design with favorable fixation stability and minimal relapse potential.

### 3.2 Biomechanical analysis results

**FIGURE 4:**
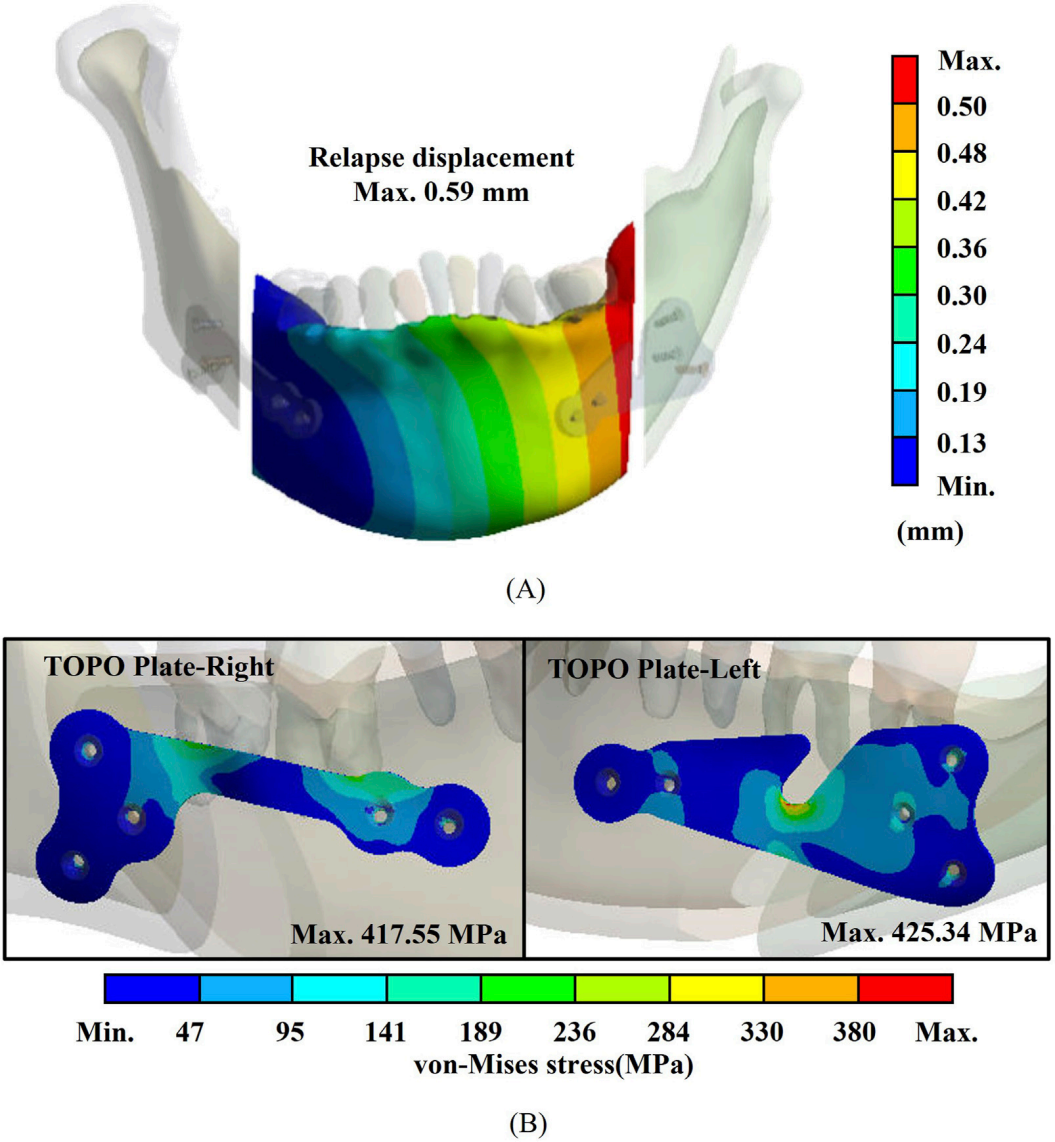
Biomechanical analysis results for the TOPO plate. **(A)** Plate stress. **(B)** Displacement of the mandibular segment.

The biomechanical analysis revealed that the maximum von Mises stress on the TOPO plate was 425.34 MPa, while the displacement of the mandibular segment was 0.59 mm. The stress observed on the plate remained well below the yield strength of Ti6Al4V (1,052 MPa) (Karolewska et al., 2020), indicating that the TOPO plate exhibits favorable fixation stability and can effectively withstand muscle traction forces and occlusal forces (Figure 4).

### 3.3 Results of static/dynamic four-point bending test

The results of the static tests showed that the right TOPO plate exhibited an average proof load of 329.90 ± 8.70 N, an average bending strength of 1,634.49 ± 43.49 N-mm, an average bending stiffness of 290.55 ± 7.75 N/mm, and an average bending structural stiffness of 302,659.38 ± 8,072.68 N-mm^2^. The left TOPO plate exhibited an average proof load of 389.85 ± 22.34 N, an average bending strength of 1949.25 ± 111.71 N-mm, an average bending stiffness of 276.14 ± 14.87 N/mm, and an average bending structural stiffness of 287,649.31 ± 15,485.52 N-mm^2^.

**FIGURE 5:**
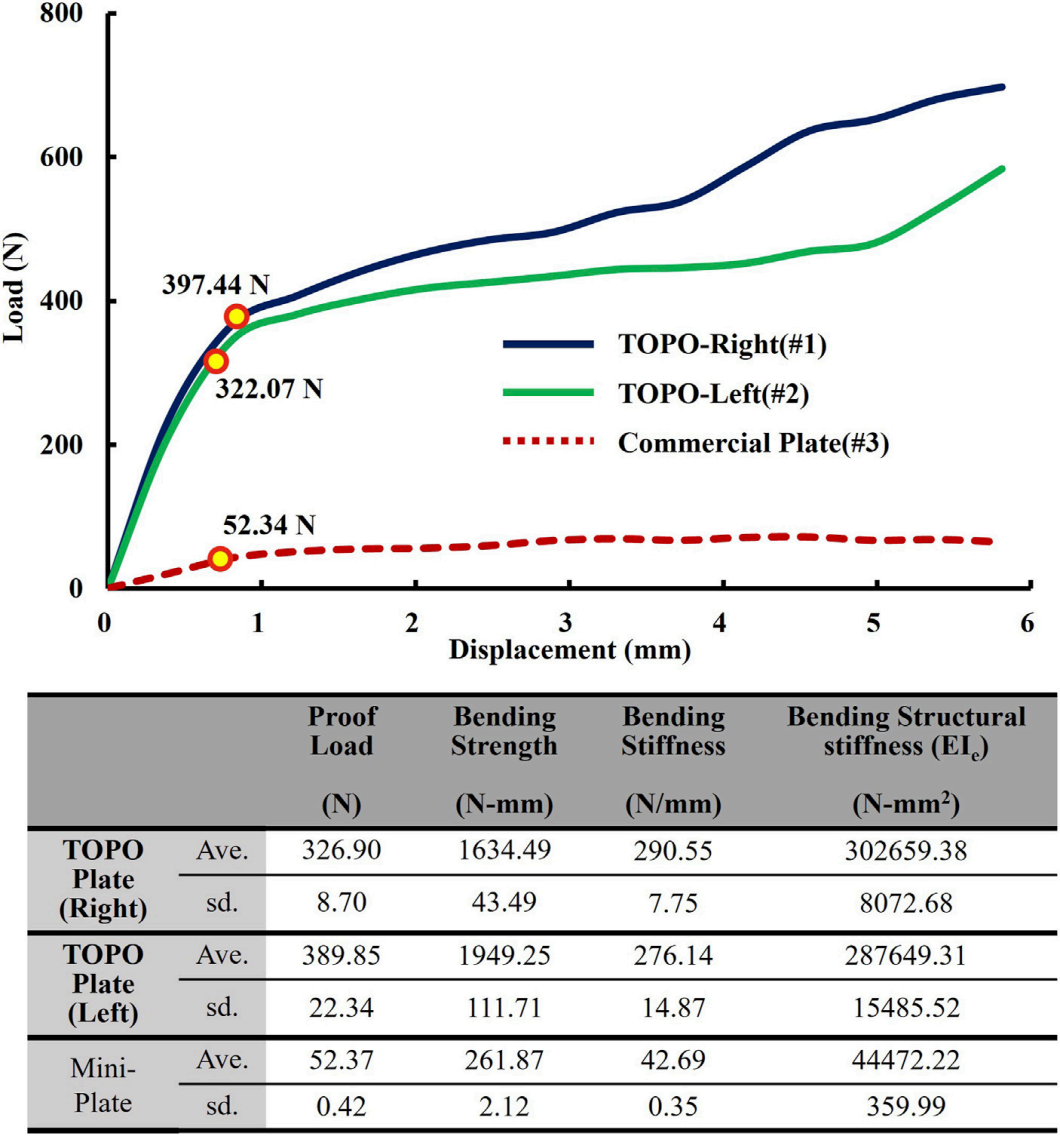
Static four-point bending test results of TOPO plate and commercial plate.

In comparison, the commercial plate exhibited an average proof load of 52.37 ± 0.42 N, an average bending strength of 261.87 ± 2.12 N-mm, an average bending stiffness of 42.69 ± 0.35 N/mm, and an average bending structural stiffness of 44,472.22 ± 359.99 N-mm^2^. The results indicated that the proof load, bending strength, bending stiffness, and bending structural stiffness of the left and right TOPO plate were significantly higher than those of the commercial plate (Figure 5).

The results of the dynamic test (Table 4) revealed that the right TOPO plate endured 1,000,000 cycles under a force of 97.46 N (25% of the proof load), whereas the left TOPO plate withstood a force of 81.73 N (25% of the proof load). In contrast, the commercial plate withstood a force of 40.85 N (78% of the proof load), demonstrating a considerably lower fatigue loading capacity than the TOPO plate.

**TABLE 4:** The results of the dynamic four bending fatigue test.

Plate	Percentage of Proof Load	Load (N)	No.	Number of cycles	Result
Right-side TOPO Plate (326.90 N)	25%	8.17–81.73 N	1	1,000,000	Pass
			2	1,000,000	Pass
			3	1,000,000	Pass
	35%	11.44–114.42 N	4	1,000,000	Pass
			5	619,708	Fail
			6	1,000,000	Pass
	45%	14.71–147.12 N	7	274,096	Fail
			8	176,001	Fail
			9	789,261	Fail
Left-side TOPO Plate (389.85 N)	25%	9.75–97.46 N	1	1,000,000	Pass
			2	1,000,000	Pass
			3	1,000,000	Pass
	35%	13.65–136.45 N	4	531,316	Fail
			5	665,309	Fail
			6	991,059	Fail
	45%	17.54–175.43 N	7	171,250	Fail
			8	143,252	Fail
			9	139,344	Fail
Commercial plate (52.37 N)	78%	4.08–40.85 N	1	1,000,000	Pass
			2	1,000,000	Pass
			3	1,000,000	Pass
	88%	4.61–46.09 N	4	247,627	Fail
			5	235,046	Fail
			6	237,658	Fail
	98%	5.13–51.32 N	7	389,294	Fail
			8	152,627	Fail
			9	145,199	Fail

### 3.4 Biomechanical test results

**FIGURE 6:**
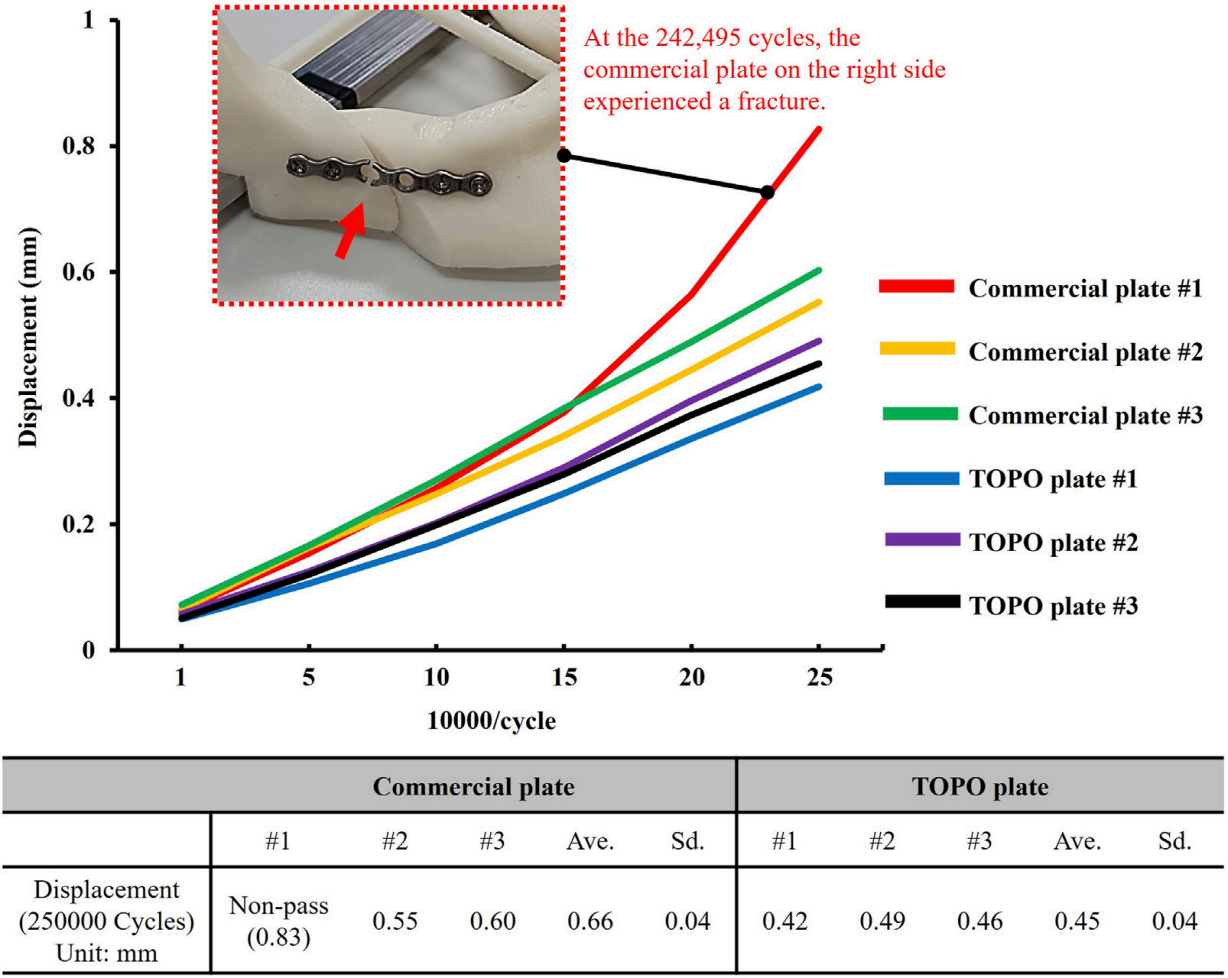
The biomechanical test results of the TOPO plate and the commercial plate.

The biomechanical test results revealed that the TOPO plate passed 250,000 loading cycles, with an average displacement of the mandible segment measuring 0.45 ± 0.04 mm. In contrast, one failed at 242,495 testing cycles among the commercial plates, and the displacement across the three commercial plates averaged 0.66 ± 0.15 mm. This highlighted the superior fixation stability of the TOPO plate compared to the commercial plate (Figure 6).

## 4 Discussion

### 4.1 Statistical validity of 30 groups hemifacial microsomia CT images for assessing mandibular asymmetry

This study randomly selected 30 sets of medical images to establish a hemifacial microsomia database and measured the mandible’s RH and WB dimensions. Medical images with the maximum, minimum, and closest-to-average RMS values of the RH and WB dimensions were chosen as the topological and parameter optimization analysis models. Although the database comprised only 30 sets of medical images, the statistical data, after normalization, demonstrated a distribution close to normal, with all samples falling within the 3σ range (Kwak and Kim, 2017), indicating the absence of outliers. This suggests that the degree of mandibular asymmetry in hemifacial microsomia may fall within a certain range. The hemifacial microsomia database and the selected analysis model hold a certain degree of representativeness. Therefore, the TOPO plate designed based on the database data is deemed suitable for the majority of hemifacial microsomia patients. Utilizing the database, this study determined the fundamental appearance of the TOPO plate. It substantiated, through relevant tests, that both the strength and fixation stability of the TOPO plate surpassed those of commercial plates.

### 4.2 Design advantages of integrating topological optimization and parameter optimization

The plate designed solely through topological optimization, referred to as a customized plate, can be tailored to meet the specific clinical needs of individual patients (Dowgierd et al., 2023). Most existing patient-specific fixation plates rely heavily on case-by-case anatomical modeling and manual or semi-automated design workflows, which limits scalability and increases the time and cost required for each individual patient. In contrast, the authors introduce a hybrid design approach that integrates topology optimization (TO) and parameter optimization (PO) to develop a general-purpose fixation plate. This method captures shared structural characteristics derived from a representative cohort of hemifacial microsomia (HFM) cases, enabling the plate to accommodate a wide range of patients without requiring fully customized designs for each case. The key innovation of this work lies in its balance between generalizability and anatomical conformity. While the overall geometry of the plate is standardized, the curvature can still be adjusted based on patient-specific CT data, ensuring an anatomically conforming fit through a streamlined digital workflow. This design paradigm offers a cost-effective, time-efficient, and clinically versatile solution that bridges the gap between fully generic and fully patient-specific implants.

Moreover, the shape of bone plates derived from topological optimization alone often appears indistinct and prone to discontinuities (Table 3). Redesigning these plates for shape and dimension relies heavily on the designer’s experience and judgment, which may compromise the plate’s structural integrity and quality. Previous studies have explored the optimization sequence (Tyflopoulos and Steinert, 2020), revealing that the design process of TO- > Redesign- > PO requires approximately only 1/6 of the optimization analysis time compared to PO- > TO- > Redesign. Although this approach increases the final product volume (with the TOPO plate being 17.1%–26.3% larger than the TO plate (Table 5)), it avoids the issues of blurred contours and discontinuous shapes that arise during redesign in the PO- > TO- > Redesign process. Therefore, this study adopts the TO- > Redesign- > PO sequence for the TOPO plate design workflow. In addition to the optimization sequence, static/dynamic four-point bending and biomechanical tests are conducted to validate the structural strength of the TOPO plate. The results demonstrate that the TOPO plate’s strength surpasses that of commercially available bone plates, ensuring its suitability for clinical applications. This method simplifies the complex workflow of the plate design process while maintaining compatibility with traditional manufacturing procedures and ensuring adequate structural strength. Moreover, the flexibility of the TOPO plate makes it more suitable for a broader patient population.

**TABLE 5:** Plate volume before and after topology optimization analysis/parameter optimization analysis.

The original volume and dimensions of the plate		
1,560 mm^3^ (L40XH26XT1.5 mm)		
Topology optimization analysis plate (TO plate) volume		
Case #1	Case #2	Case #3
480.10 mm^3^	427.23 mm^3^	471.17 mm^3^
Topology and parameter optimization analysis plate (TOPO plate) volume		
579.30 mm^3^		

### 4.3 Sensitivity analysis of parameter optimization

**FIGURE 7:**
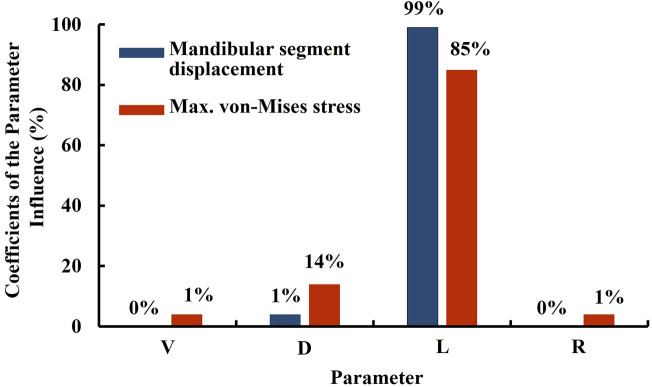
Sensitivity analysis results of the TOPO plate.

Evaluating the influence of model input parameters on output results can be conducted using sensitivity analysis. This approach helps identify which parameters significantly affect the outcomes, thereby determining the key parameters of the plate (Moradi et al., 2021; Ovesy et al., 2019). These key parameters should remain unchanged under any specific conditions to maintain structural strength. However, to accommodate manufacturing requirements, parameters with less impact on the results may be adjusted as needed, aiming to minimize their influence on the parameter optimization outcome (Figure 7). The sensitivity parameters selected in this study are the right-side plate width (V), left-side plate groove width (D), left-side plate groove depth (L), and the distal fillet on both sides of the plate (R). The response variables measured are the displacement of the mandible (Displacement) and the maximum von Mises stress on the plate (max. von Mises stress). The same methodology and settings were used for both sensitivity analysis and parameter optimization. The results indicate that when the response variable in sensitivity analysis is the displacement of the mandibular segment, the influence proportion is 99% for the left-side plate groove depth (L), 1% for the left-side plate groove width (D), and 0% for both the right-side plate width (V) and the distal fillet (R). When setting the response as the maximum von Mises stress, the influence proportion is 85% for the l left-side plate groove depth (L), 14% for the left-side plate groove width (D), while the influence proportions of the right-side plate width (V), and 1% each for the right-side plate width (V) and the distal fillet (R). These findings indicate that the primary key parameter, regardless of the response variables, is the left-side plate groove depth (L), followed by the left-side plate groove width (D), with minimal impact from the right-side plate width (V) and the distal fillet (R). If future adjustments to the bone plate structural parameters are necessary, priority should be given to modifying the right-side plate width (V) and the distal fillet of both sides of the plate (R) parameters to avoid impacting the structural strength and fixation stability of the bone plate. Conversely, if it is necessary to adjust the left-side plate groove depth (L) and the left-side plate groove width (D), biomechanical analysis or *in vitro* functional testing should be conducted to reconfirm the structural strength and fixation stability of the bone plate.

### 4.4 Advantages of TOPO plate in static/dynamic four-point testing

The ASTM F382 regulation test is a standard specification and test method for metallic bone plates, defining the four-point bending fatigue test to compare the fatigue performance of different plate designs (Schorler et al., 2018). This method determines structural strength and fatigue life of bone plates under specific maximum bending moments, crucial for evaluating bone plates intended for internal fixation. These plates must be substantially compared to commercially available plates before being marketed. In this study, the ASTM F382 standard testing was employed to evaluate the performance of TOPO plates. Static four-point bending tests revealed that both left and right TOPO plates exhibited higher average proof load, bending strength, bending stiffness, and bending structural stiffness than commercial plates. This superiority can be attributed to the increased volume of the TOPO plate and the resulting higher cross-sectional moment of inertia (I) compared to commercial plates, directly enhancing the bending structural stiffness. Dynamic four-point bending tests further demonstrated that TOPO plates surpassed commercial plates, with fatigue loads (proof loads) approximately twice as high. The fatigue load limit, represented by bending strength (Ph/2, where P is the percentage of proof load passed in fatigue testing, and h is the loading span distance of 10 mm), were 408.65 N-mm and 487.3 N-mm for the right and left TOPO plates, respectively, compared to 204.25 N-mm for the commercial plate. These results from static and dynamic four-point bending tests confirmed that both left and right TOPO plates have superior structural strength compared to commercial plates. The TOPO plate demonstrates safety and efficacy, meeting the requirements for market certification. Supporting literature employing the same machine and parameters has reported porosity levels as low as 0.41%–0.06% (Wang et al., 2020), indicating a very low internal porosity, with a microstructure comparable to conventional cast titanium (Brachet et al., 2023). Such minimal porosity is not expected to significantly impact mechanical performance (Prządka et al., 2025). Whether internal defects affect mechanical performance can also be indirectly assessed through the static and dynamic four-point bending tests and biomechanical tests conducted in this study. The TOPO plate exhibited no signs of brittle fracture or catastrophic failure, but instead showed ductile deformation behavior, resulting in only minor displacement of the mandibular segment in BSSO reconstruction.

### 4.5 Biomechanical performance of the TOPO plate

While static and dynamic four-point bending tests can verify whether the structural strength of the TOPO plate meets market requirements, these tests are insufficient to simulate the stability of plate fixation or its ability to resist relapse. To address this, this study employs biomechanical testing to confirm the biomechanical performance of the plate when fixed to the mandible. Biomechanical testing simulating 6 months of occlusion reveals concerns regarding the commercial plate’s durability, as one set of plates experienced fracture (Gellrich et al., 2004). This aligns with existing literature, which highlights the insufficient strength of commercial plates and the potential risk of fracture (Seol et al., 2014). Clinically, this issue is challenging to remedy, requiring removal of the fractured plate and loose screws and re-fixation of the mandibular segment. This intricate process cannot guarantee stable fixation in the secondary surgery (Seol et al., 2014; Kadam, 2019). These ensuing complications are burdensome for patients. Ignoring the fractured commercial plates, it was observed that after exceeding 50,000 loading cycles, the displacement of the mandibular segment with commercial plates increased significantly. This indicates insufficient early stability of the commercial plates, potentially affecting the healing of the mandibular segment (Foster et al., 2021). Relevant literature emphasizes that stability is crucial for fracture healing, with surgical interventions such as internal or external fixation enhancing this stability. Adequate mechanical stability promotes the differentiation of mesenchymal stem cells (MSCs) towards the bone interface, positively impacting bone healing (Foster et al., 2021). Minimizing micromotion at the bone interface plays a critical role in accelerating bone healing. During the early healing phase, the TOPO plate provides stable fixation that maintains consistent contact at the bone interface, thereby creating a favorable environment for bone regeneration (Raffa et al., 2019). Biomechanical testing showed a displacement of 0.45 mm for the TOPO plate compared to 0.66 mm for the commercial plate. Although the difference of 0.21 mm is not statistically significant, the TOPO plate offers a distinct advantage in promoting bone interface integration by adhering to the principle of minimizing interfacial movement.

### 4.6 Research limitations

This study has several limitations. The TOPO plate developed in this study is specifically intended for use in mandibular reconstruction of patients with hemifacial microsomia (HFM). The design is based on morphological patterns and biomechanical considerations derived exclusively from an HFM patient population. As such, the TOPO plate may not be suitable for mandibular reconstruction in patients with other craniofacial conditions, such as Parry-Romberg syndrome, post-traumatic deformities, or oncologic resections, where the etiology, anatomical characteristics, and reconstructive requirements differ significantly. Future studies are needed to explore the applicability of the TOPO design methodology in broader clinical contexts. Although the current HFM image database (30 cases) suggests that mandibular asymmetry in HFM patients may be confined to a limited and clinically definable range, the inclusion of a more demographically diverse sample set would enhance the robustness and adaptability of the design. Future studies should incorporate expanded datasets across different severity levels, age groups, and gender categories to further validate and refine the applicability of the current design model. While relevant *in vitro* functional tests have confirmed that the structural strength and biomechanical behavior of the TOPO plate meet clinical requirements, further validation is necessary. Future work should include a greater number of repeated experiments to allow for more rigorous statistical evaluation. Increasing the sample size will further enhance the reproducibility and reliability of the mechanical performance data of the TOPO plate design. Specifically, the safety and efficacy of the TOPO plate should be tested in more complex biological environments. As a continuation of this research, a future preclinical *in vivo* animal study is planned to evaluate the mechanical stability and clinical applicability of the TOPO plate under physiological conditions. A porcine model will be employed, in which patient-specific TOPO plates will be used to reconstruct mandibular defects created by BSSO osteotomies. Postoperative evaluations will be conducted at multiple time points under mechanical loading, with CT imaging used to quantitatively assess implant stability. This animal study will serve as a critical step in validating the functional efficacy and translational potential of the TOPO plate design prior to clinical application. It is proposed that the TOPO plate is appropriate for patients with an RMS value less than or equal to 37.62. This threshold reflects the current upper limit validated through mechanical testing and biomechanical analyses. Should future expansions of the database include patients with higher RMS values, the applicability threshold may be redefined accordingly. However, any adjustment to the RMS limit would require rigorous re-analysis and comprehensive mechanical validation before clinical implementation.

## 5 Conclusion

This study aimed to develop the TOPO plate by integrating topology and parameter optimization. Through a database of hemifacial microsomia cases, the degree of mandibular deviation was confirmed, and the TOPO plate was devised to achieve a lightweight and stable structure, reducing the risk of relapse.

In static and dynamic four-point bending tests, the structural strength of the TOPO plate (Right: ave. proof load of 326.9 N, left: ave. proof load of 389.85 N) significantly exceeded that of commercial plates (ave. proof load of 52.37 N). The TOPO plate also demonstrated superior resistance to fatigue loads (Right: pass loading of 81.73 N, left: pass loading of 97.46 N) compared to commercial plates (pass loading of 40.85 N).

Biomechanical tests further confirmed that the TOPO plate limits the displacement of mandibular movement (ave. 0.45 mm), providing favorable stability for fixation. Overall, the TOPO plate meets the biomechanical requirements for mandibular behavior and complies with market demands, demonstrating its potential as a superior solution for mandibular fixation.

## Data Availability

The original contributions presented in the study are included in the article/supplementary material, further inquiries can be directed to the corresponding author.
